# Profiling Taste and Aroma Compound Metabolism during Apricot Fruit Development and Ripening

**DOI:** 10.3390/ijms17070998

**Published:** 2016-06-24

**Authors:** Wanpeng Xi, Huiwen Zheng, Qiuyun Zhang, Wenhui Li

**Affiliations:** 1 College of Horticulture and Landscape Architecture, Southwest University, Chongqing 400716, China; zhenghuiwen553@foxmail.com (H.Z.); swuzqy@163.com (Q.Z.); 2 Key Laboratory of Horticulture Science for Southern Mountainous Regions, Ministry of Education, Chongqing 400715, China; 3 National Fruit Tree Germplasm Repository, Xinjiang Academy of Agricultural Sciences, Luntai, Xinjiang 841600, China; lwh-2003@163.com

**Keywords:** apricot, sugars, organic acids, volatiles, flavor, metabolites, fruit quality

## Abstract

Sugars, organic acids and volatiles of apricot were determined by HPLC and GC-MS during fruit development and ripening, and the key taste and aroma components were identified by integrating flavor compound contents with consumers’ evaluation. Sucrose and glucose were the major sugars in apricot fruit. The contents of all sugars increased rapidly, and the accumulation pattern of sugars converted from glucose-predominated to sucrose-predominated during fruit development and ripening. Sucrose synthase (SS), sorbitol oxidase (SO) and sorbitol dehydrogenase (SDH) are under tight developmental control and they might play important roles in sugar accumulation. Almost all organic acids identified increased during early development and then decrease rapidly. During early development, fruit mainly accumulated quinate and malate, with the increase of citrate after maturation, and quinate, malate and citrate were the predominant organic acids at the ripening stage. The odor activity values (OAV) of aroma volatiles showed that 18 aroma compounds were the characteristic components of apricot fruit. Aldehydes and terpenes decreased significantly during the whole development period, whereas lactones and apocarotenoids significantly increased with fruit ripening. The partial least squares regression (PLSR) results revealed that β-ionone, γ-decalactone, sucrose and citrate are the key characteristic flavor factors contributing to consumer acceptance. Carotenoid cleavage dioxygenases (CCD) may be involved in β-ionone formation in apricot fruit.

## 1. Introduction

Apricot (*Prunus armeniaca* L.) is an important Rosaceae family fruit crop, and its fruits contain many nutrients and phytochemicals that contribute to a healthy diet. Generally, apricots are appreciated by consumers for their unique flavor. In fact, flavor is not only a fruit quality factor that determines consumer preference, but also serves as a crucial clue to signal nutrient makeup to humans [1]. Fruit flavor is derived from a combination of taste and aroma [2,3]. The taste of apricot primarily depends on sugars and organic acids, whereas the aroma depends on a large number of volatile organic compounds (VOCs).

Flavor compound accumulation during fruit development has been widely studied in different species. The composition and content of sugars, organic acids and aroma volatiles usually changes with fruit development. Soluble sugars reach the peak at maturation or ripening [4]. Organic acids usually accumulate at the early stages of fruit development and are used as respiratory substrates during fruit ripening, and subsequently to their lowest levels in ripe fruit [5,6]. For aroma compounds, the fruity odor compounds such as esters will increase significantly during the late fruit development, while the green odor such as hexenal rapidly decrease. In most fruit, glucose and fructose form the major portion of soluble sugars, whereas in peach, litchi and mandarin, sucrose is the predominant sugar. Fructose is by far the major sugar in apple. For organic acids, malic acid and citric acid predominate quantitatively in most fruits. The final organic acid concentration in ripe fruit is determined by the balance of organic acid biosynthesis, degradation and vacuolar storage.

However, flavor compounds accumulation patterns and concentrations differ between species even cultivars. To date, the effect of development and ripening on flavor compound accumulation has been extensively investigated in tomato [7], peach [8,9] plum [10], strawberry [11], gojiberry [12], sweet orange [13], melon [14], pineapple [15] and grape [16]. So far, the current studies on fruit flavor have focused on specific flavor compounds, one developmental stage, individual enzymes, or a single variety. Only a few detailed studies addressing sugars, organic acids and aromas as a whole and as a dynamic system are available in the literature. Up to now, chemical changes [17] and aroma–related genes during ripening of apricot fruit [18], as well as a rapid method of determination of sugars and organic acids [19] have been mentioned, however, the flavor profile of apricot is not fully clear. The knowledge about how flavor compounds shape underlying development remains to be unraveled. As the indispensable information for improvement strategies of flavor quality, the main flavor compounds in apricot fruit are still not yet identified.

Thus, in the study, we intended to firstly integrate sugars, organic acids and aroma volatile information and to provide a unique, extensive vision of developmental dynamics of flavor compound metabolism in apricot. To this end, a large fraction of the metabolites and enzymes involved in metabolism of these flavor compounds were assayed within five genotypes at different times of fruit development to delineate the complex process of flavor shaping and to reveal the mechanism underlying this process. Additionally, the key taste and aroma components in fruit were identified. These results will provide a powerful resource for uncovering key components in the regulation of metabolic networks and give the important information for consumer-oriented breeding.

## 2. Results

### 2.1. Quality Traits of Apricot during Development and Ripening

**Figure 1:**
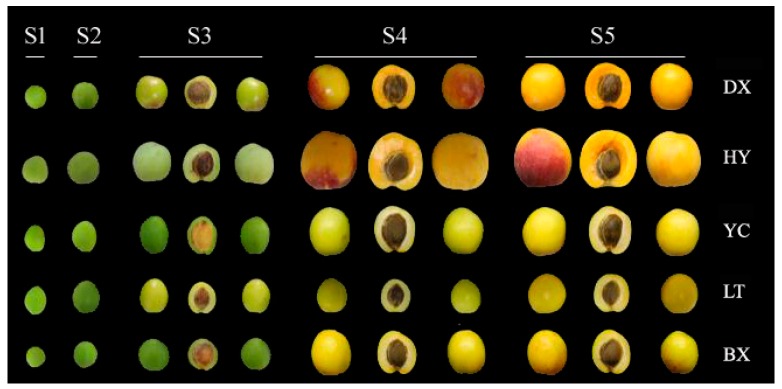
Morphology of apricot fruit during development and ripening. The sampling time for each stage is listed in Table 1. They are represented by immature green (S1); maturation green (S2); turning (S3); green ripe (S4) and full ripe (S5). Cultivar abbreviations (as listed in Table 1) are shown at right.

**Figure 2:**
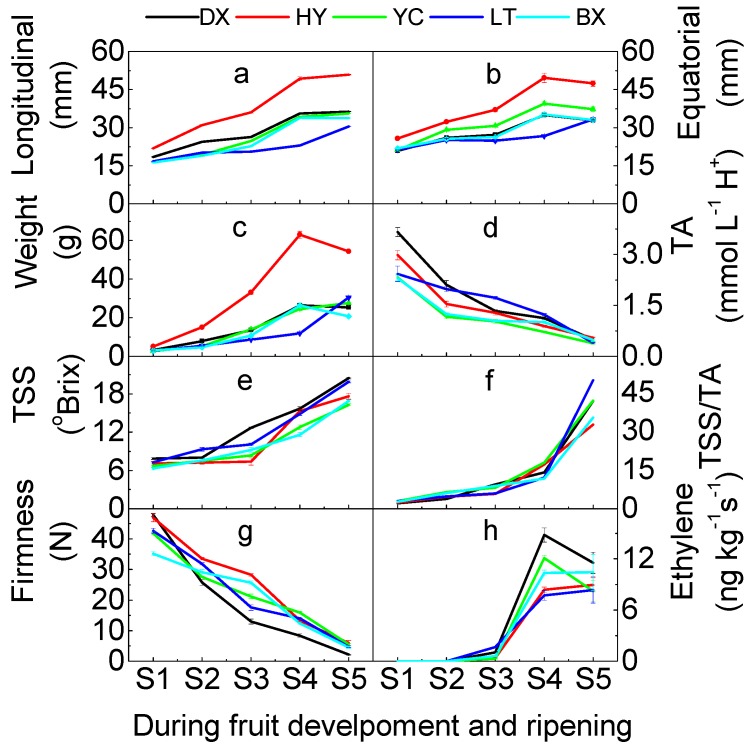
Fruit quality traits of apricots during development and ripening. (**a**) Fruit longitudinal diameter; (**b**) Fruit equatorial diameter; (**c**) Weight of single fruit; (**d**) TA, titratable acid; (**e**) TSS, total soluble solid; (**f**) TSS/TA, the ratio of TSS and TA; (**g**) Fruit firmness; (**h**) Ethylene production. All data are expressed as means ± standard error of triplicate samples.

Fruits were classified into five different stages (immature green, S1, maturation green, S2; turning, S3; green ripe, S4; and full ripe, S5) and picked according to peel color (Table 1 and Figure 1). Skin color changed dramatically between maturity stages and ripening. The longitudinal and equatorial diameters of cultivars tested varied from 16.25 to 50.85 mm and 20.83 to 49.55 mm during the development and ripening period, respectively (Figure 2a,b), while the weight increased from 2.6 to 54.35 g. Hongyuxing (HY) had the largest two diameters and fruit weight (Figure 2c), which presented the large fruit shape, and Danxing (DX) and Yechengheiyeyxing (YC) were the medium-sized fruit cultivars, Luntaixiaobaixing (LT) and Baixing (BX) were the small fruit cultivars. The development of fruits tested was characterized by cell enlargement (0–27 or 32 days), rapid enlargement (0–57 or 91 days) and a period of retarded enlargement (74–101 days).

With the development and ripening, the titratable acid (TA) level decreased to the lowest point (<0.55 mmol·L^−1^ H^+^) at the full-ripe stage. Yechengheyexing (YC) and Luntaixiaobaixing presented low TA level at ripe stage (<0.4 mmol·L^−1^ H^+^), they are low acid cultivars (Figure 2d). In contrast, the total soluble solids (TSS) of fruit increased from 6.7 to 20.47 Brix, TSS of all tested cultivars increased rapidly after turning stage (Figure 2e). For almost all cultivars tested, the TSS increased to double the amount of turning stage at the full-ripe stage, and Danxing (DX) had the highest TSS. A similar trend in change was observed in the ratio of TSS/TA (Figure 2f), the index increased slowly from 2.15 to 6.46 during early development period (S1 to S2), and then increased from 5.78 to 17.78 during the middle period (S3 to S4), at last increased rapidly from 17.78 to 49.75 (S4 to S5) during the ripening period.

Fruit softened rapidly during development and ripening (Figure 2g). The firmness of fruit for all cultivars tested declined from 47.83 to 2.15 N, and the firmness of all fruit tested was less than 6 N at the full-ripe stage. Danxing (DX) had the lowest firmness of the tested cultivars (2.15 N). The levels of ethylene production increased from stage 1 (S1) to stage 4 (S4), then decreased at the stage 5 (S5) (Figure 2h), and an obvious peak of ethylene were observed at the stage 4 (S4) for all tested fruits (8.24–11.55 ng·kg^−1^·s^−1^).

### 2.2. Changes in Sugars during Fruit Development and Ripening

**Figure 3:**
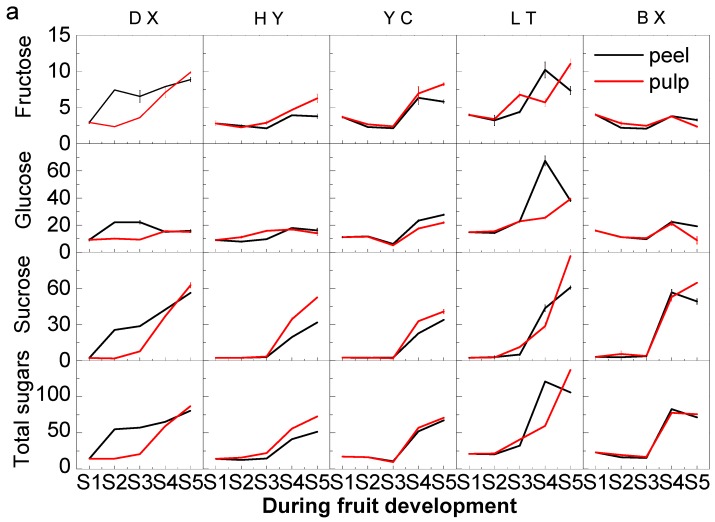

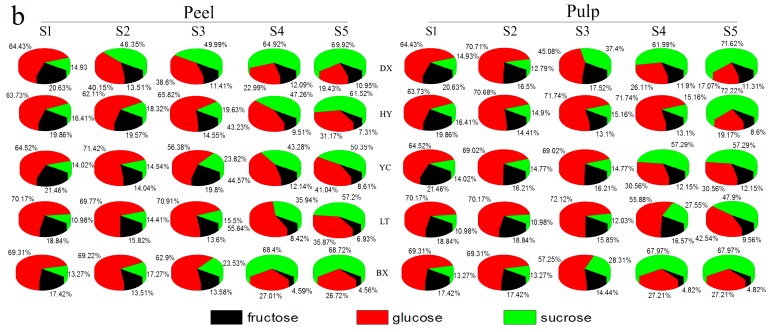
Sugar content (mg/g fresh weight, FW) and ratio in apricot fruit during development and ripening. (**a**) Changes in soluble sugar content; (**b**) The ratio of individual sugar content to total sugar content. Two capital letters in the middle are the cultivars shown in Table 1. All data are expressed as means ± standard error of triplicate samples.

As shown in Figure 3a, all sugars including fructose, glucose, sucrose and total sugars kept increasing during the whole development period. From the turning stage (S3) to the full-ripe stage (S5), the sugar contents increased especially steeply. Trace sorbitol was detected in fruit tested at all developmental stages. Even though no significant differences of sugar content during the early development (from S1 to S2) were found in all tested fruits, significantly higher fructose was observed in Danxing (DX), Luntaixiaobaixing (LT) and Baixing than those in Hongyu (HY) and Yechengheiyexing (YC) during ripening (from S3 to S5). The content of all sugars identified and total sugars in Luntaixiaobaixing (LT) were found significantly higher than those in other cultivars during ripening. Though higher contents of fructose, glucose, sucrose and total sugars were found in Danxing (DX), Yechengheiyexing (YC) and Luntaixiaobaixing (LT) peels than their pulps during the whole development period, significantly lower level of sugars were found in Hongyuxing (HY) pulps than those in its peels. At the same time, no marked difference of sugar content was found between Bainxin peels and pulps during the whole development period. Therefore, the difference of sugar contents between peels and pulps may dependent on the cultivar.

As for the ratio of sugars (Figure 3b), on the whole, ratio of glucose in fruit decreased significantly during the whole development period, it decreased from 70.17% to 19.43% in peels and 70.17% to 19.17% in pulps of tested cultivars, respectively. However, glucose was the predominant sugar during the early development (from S1 to S3) and still accounted for 38.6%–71.42% of total sugar in peels and 45.08%–70.68% of total sugar in pulps. Similar to glucose, the ratio of fructose also decreased from 21.46% to 4.56% in peels and from 21.46% to 4.82% in pulps during the whole development period. As the second major sugar at the early stage, fructose accounted for 11.41%–21.46% of total sugar in peels and 13.1%–21.46% of total sugar in pulps. However, the ratio of sucrose increased from 10.98% to 69.92% in peels of tested cultivars and 10.98% to 72.22% in pulps of tested cultivars during the whole development period, and become the major sugar in all cultivars tested during ripening (from S3 to S5), accounting for 35.94%–69.92% of total sugars in peels and 27.55%–71.62% of total sugars. Glucose become the second major sugar during ripening, it represented 19.43%–55.64% of total sugar in peels and 19.17%–55.88% in pulps of cultivars tested. During the ripening period, fructose was found at the lowest level of all tested sugars, only accounting for 4.56%–12.14% in peels and 4.82%–16.57% in pulps of cultivars tested.

### 2.3. Changes in Organic Acids during Fruit Development and Ripening

**Figure 4:**
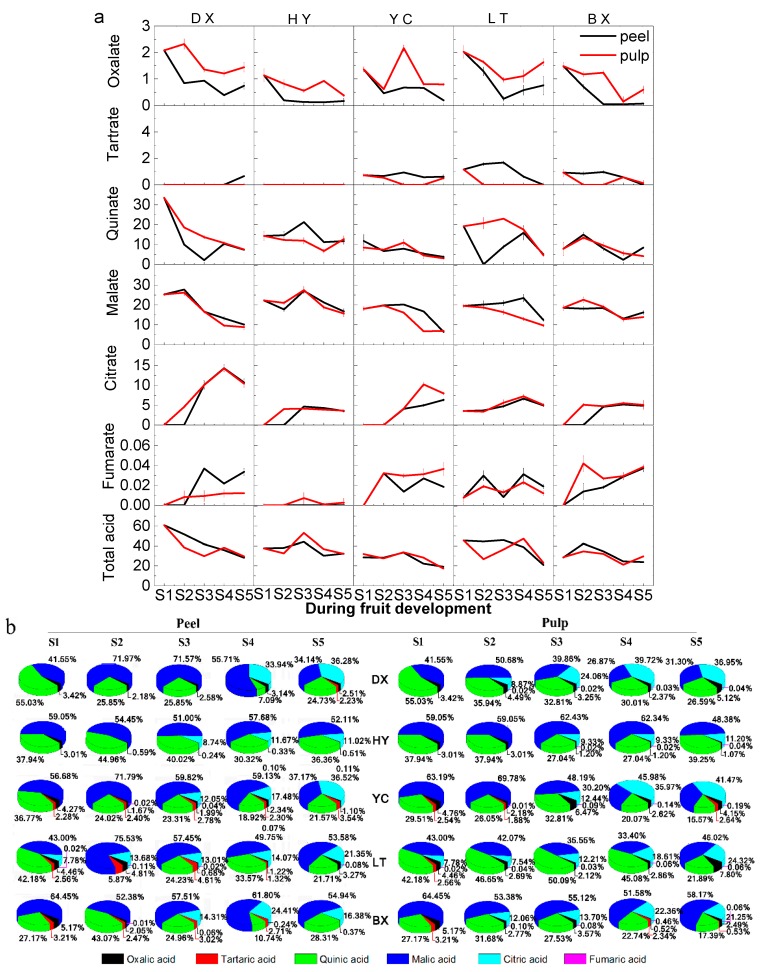
Organic acid content (mg/g FW) and ratio in apricot fruit during development and ripening. (**a**) Changes in organic acids; (**b**) The ratio of individual organic acid content to total organic acids content. Two capital letters in the middle are the cultivars shown in Table 1. All data are expressed as means ± standard error of triplicate samples.

As shown in Figure 4a, all organic acids including oxalate, tartrate, quinate, malate, citrate, fumarate and total organic acids mostly increased during the early stages of fruit development (from S1 to S3) and decreased until fruits were full-ripe (from S4 to S5). Oxalate, tartrate, quinate, malate, citrate, and fumarate decreased from 2.07 to 0.07 mg/g FW, from 1.56 to 0.12 mg/g FW, from 33.47 to 2.98 mg/g FW, from 27.72 to 6.44 mg/g FW, from 10.22–3.53 mg/g FW and from 0.03–0.01 mg/g FW, respectively. Quinate, malate and citrate were the predominant organic acids throughout the whole fruit development and ripening period. Oxalate and tartrate were found at lower levels, while trace fumarate was also detected in the fruit of cultivars tested during the whole development period.

The content of quinate in HY, YH and LT increased to the peak at the turning stage of fruit development, whereas BX accumulated the largest amount of quinate at stage 2 (S2), then decreased to the lowest point at the full-ripe stage (S5). The level of quinate in DX almost kept deceasing throughout the whole development period. Except for HY, higher level of quinate in pulps were found than in peels. DX had the highest quinate content of all tested cultivars. YC and BX presented significantly lower level of quinate than other cultivars tested. Malate in DX, YC and BX increased to the highest level at stage 2 (S2), and then kept decreasing. Malate in DX and LT reached the highest level at stage 3 (S3) and stage 4 (S4). Higher level of malate in peels of DX, YC and LT were observed than in their pulps. A significantly lower level of malate was found in YC than other cultivars tested. The level of citrate kept increasing until stage 3 (S3) or stage 4 (S4) of fruit development, and then decreased at the full-ripe stage. HY presented the lowest level of citrate than other cultivars tested. Except for YC, oxalate generally decreased throughout the whole development period, and the lowest level of oxalate was found in HY. Significantly higher levels of oxalate were found in pulps than in peels. Tartrate was only detected in YC, LT, BX and DX peels, and the content of tartrate in peels tested increased to the highest level at stage 3 (S3), and then declined. Higher levels of tartrate in peels were observed than in pulps. Total acid had a similar trend to that of quinate. DX had significantly higher organic acids identified than other cultivars.

As for the ratio (Figure 4b), quinate, malate and citrate are the predominant organic acids in apricot regardless of developmental stage. Malate is the first major organic acid in apricot. The ratio of malate in fruit decreased from 71.97% to 34.14% in peels and 69.78% to 21.25% in pulps of cultivars tested, respectively. The ratio of quinate also decreased from 55.03% to 7.09% of total organic acids in peels and from 55.03% to 15.57% in pulps during development and ripening, respectively. However, the ratio of citrate increased significantly from 0% to 36.52% in peels and 0% to 45.98%, respectively. Quinate and malate account for more than 85% of total organic acids at the early stage, they are the major organic acids of fruit tested during early development. However, with the increase in the ratio of citrate in fruit, quinate, malate and citrate occupied 93% of total organic acids in both peels and pulps. The ratio of oxalate and tartrate to total organic acids was only 0%–6.47% and 0%–5.87%, respectively. From the ratio of individual organic acid to total organic acids, YC, LT and BX had a richer variety of organic acids than DX and HY.

### 2.4. Changes in Aroma Volatiles during Development and Ripening

A total of 46 aroma compounds were identified, including eight aldehydes, five alcohols, seven esters, five norisoprenoids, eight lactones, ten terpenes and six acids (Table S2). Among them, 18 aroma compounds, including three aldehydes (hexanal, (*Z*)-3-hexenal, (*E*,*Z*)-2,6-nonadienal), three apocarotenoids (β-damascenone, β-ionone, dihydro-β-ionone), five lactones (γ-octalactone, δ-octalactone, γ-decalactone, δ-decalactone, γ-dodecalactone), five terpenes (β-myrcene, linalool, α-terpineol, geraniol, limonene) and two esters (hexyl acetate, (*Z*)-3-hexenyl acetate), were the major aroma compounds in apricots by odor activity values (OVA) method. The OVAs of these 18 aroma compounds were more than 1 (Table 2). γ-decalactone was the highest OVA compound in all cultivars tested, it ranged from 145 to 319 with the highest OVA in LT peel. β-ionone was the second major aroma compound in apricot fruit tested. It ranged from 40 to 506. The OVA of β-ionone in LT and BX were 4.65-16.09 times greater than those in other cultivars. γ-dodecalactone was the third major aroma compound and it ranged from 37.21 to 160 with the highest level in LT peel. High OVAs of β-damascenone (180) and hexyl acetate (183), (*E*,*Z*)-2,6-nonadienal (319), and α-terpineol (103) were found in DX, whereas high OVAs of β-ionone (506), γ-dodecalactone (160), γ-decalactone (319) and hexyl acetate (183) was found in LT. Significantly higher OVAs were observed in peels than pulps for all cultivars tested.

**Figure 5:**
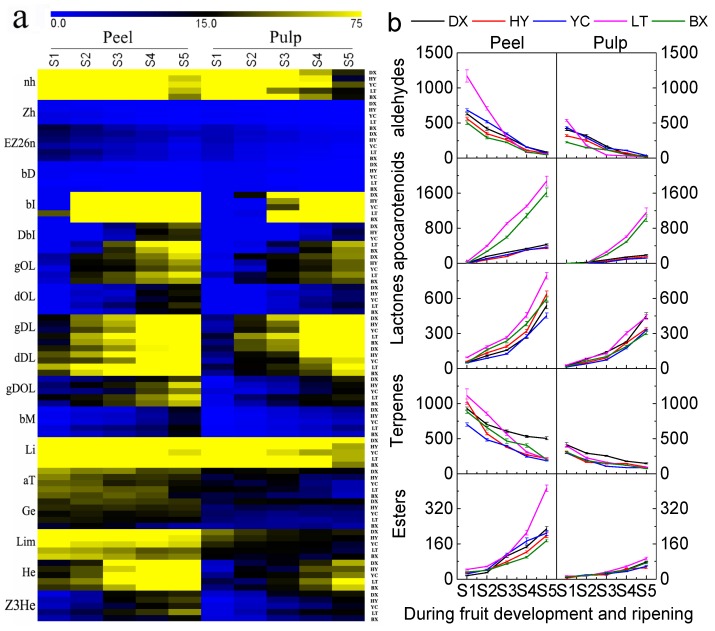
Volatile compound contents (μg/kg FW) in apricot fruit during development and ripening. (**a**) The left letters represent the aroma compounds in Table 2, the right double capital letters represent the cultivars in Table 1; (**b**) Total contents of each type of aroma compound during fruit development and ripening. The top double capital letters present the cultivars in Table 1. All data are expressed as means ± standard error of triplicate samples.

Changes in 18 major aroma compounds during the whole development period are given in Figure 5. The contents of hexanal, (*Z*)-3-hexenal and (*E*,*Z*)-2,6-nonadienal decreased rapidly throughout the whole development period (Figure 5a). The total aldehydes decreased from 1170 to 47.8 μg/kg FW. Significantly higher aldehydes were observed in peels than in pulps. As the most abundant aroma compounds in apricot, the contents of β-damascenone, β-ionone and dihydro-β-ionone increased dramatically during fruit ripening, especially the major apocarotenoids. The content of β-ionone increased from 155 to 1875 μg/kg FW in peels and from 30 to 1026 μg/kg FW in pulps of all cultivars tested. It is very interesting that the content of β-ionone, dihydro-β-ionone and total apocarotenoids in LT and BX were significantly higher than those in other cultivars, especially the β-ionone. Similarly, the contents of apocarotenoids in peels were significantly higher than those in the pulps of cultivars tested. Lactones were the second most abundant aroma compounds, and γ-decalactone was the predominant lactone, it ranged from 17.14 to 350.71 μg/kg FW in peels and 5–277.14 μg/kg FW in pulps, respectively. BX had the highest γ-decalactone in cultivars tested. Total lactones also increased rapidly during the ripening period. Conversely, β-myrcene, linalool, α-terpineol, geraniol and limonene all decreased through the ripening period. Linalool was the predominant terpene in these fruits, and it decreased from 987 to 122 μg/kg FW in peels and from 356 to 45 μg/kg FW in pulps during the whole development period. DX had the highest level of linalool and total terpenes during fruit ripening. Two major esters, hexyl acetate and (*Z*)-3-hexenyl acetate, increased during the whole development period, especially as the predominant, ester hexyl acetate increased from 13 to 365 μg/kg FW in peels and 3 to 76 μg/kg FW in pulps, respectively. LT peel had the highest level of total esters. The contents of total aldehydes and terpenes in all cultivars decreased rapidly during fruit development and ripening, while the contents of total lactones and apocarotenoids kept increasing during this period (Figure 5b). It is interesting that the contents of individual and total apocarotenoids in LT and BX were significantly higher than those in other cultivars in both peels and pulps (Figure 5). On the whole, peels presented significantly individual or total aroma volatiles than pulps.

### 2.5. Effect of Flavor Compounds and Ripening on Consumer Acceptance

**Figure 6:**
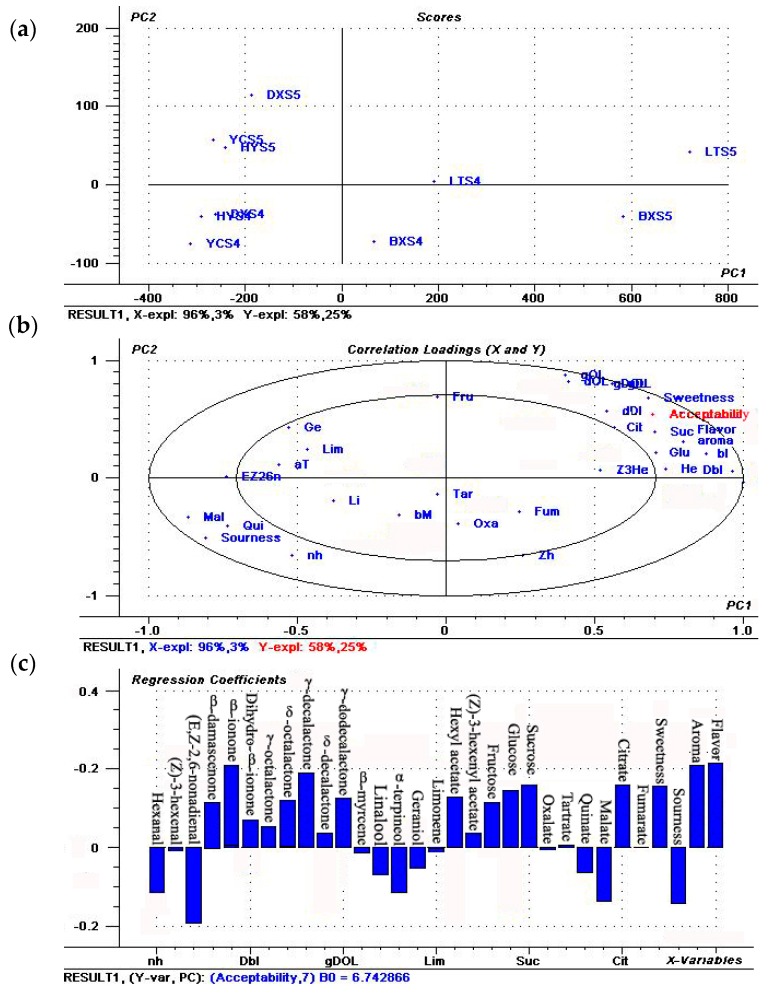
The PLSR model of flavor compounds and sensory attributes of apricot fruit. (**a**) Scores plot, the first two capital letters represent the genotypes described in Table 1 and Tables S4 and S5 represent the last ripening stage of apricot fruit, blue word represents the independent variable, and red word represents the dependent variable; (**b**) Correlation loadings. Flavor compound codes and sensory parameters are indicated in Table 2 and Table 3; (**c**) Regression coefficients from the PLSR model of flavor compounds and sensory attributes (X variables) versus consumer acceptances (Y variable) for apricot fruit during ripening.

The result showed that flavor compounds and sensory attributes account for 83% of total variability in consumer acceptability (Figure 6a). Cultivar and development stage appeared to be the main differentiating factors. Fruits were clearly distributed in different locations in the score plot, based on the stage of cultivars or development variable. The DX, HY and YC were located on the lower left side of the PC1, and LT and BX on the right side of the PC1, while cultivar-stage points were clearly separated from the cultivar-stage points (Figure 6a). Figure 6b suggested that cultivar—stage DXS4, HYS4 and YCS4 were not the best fruit for consumers mainly due to high level of n-hexanal, malic acid, organic acid content and high sourness; whereas LTS5, BXS5, especially LTS5, were the best mainly due to high contents of β-ionone, γ-decalactone, sucrose, citrate, sweetness, aroma and flavor (Figure 6b,c). These results suggested that excellent flavor quality and consumer acceptability have a positive relationship with the levels of these above parameters.

### 2.6. Enzymes Involved in Flavor Compound Metabolism during Development and Ripening

**Figure 7:**
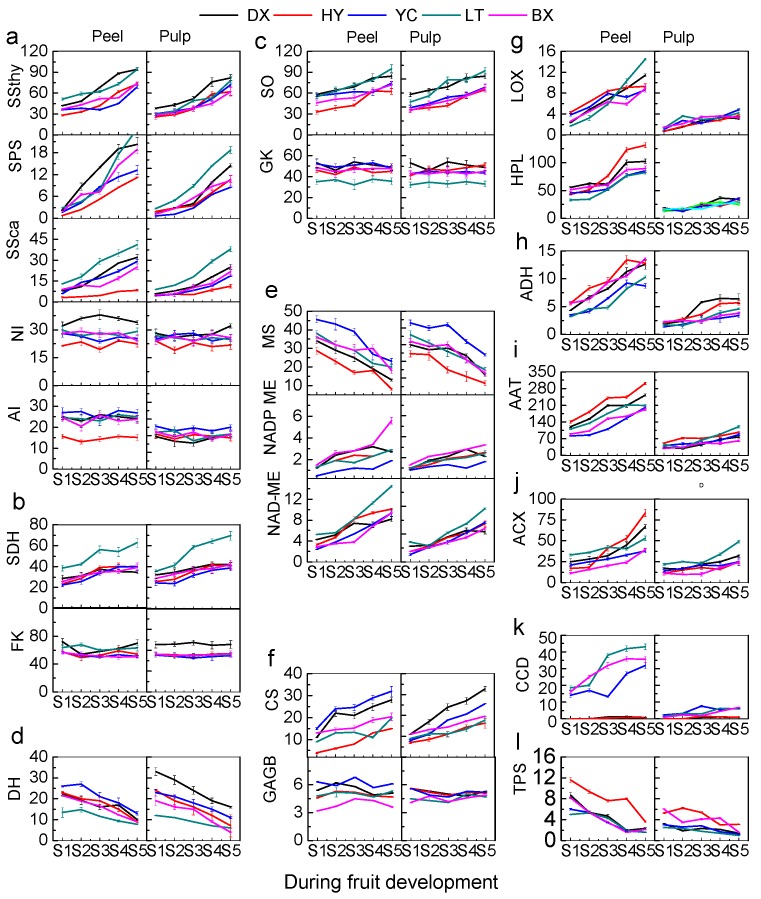
The flavor compound-related enzyme activities (U/mg protein) of apricot fruit during development and ripening. (**a**) SSthy, sucrose synthase for synthesis direction, SPS, sucrose phosphatesynthase, SSca, sucrose synthase for degradation direction, NI, neutral invertase, AI, acid invertase; (**b**) SDH, sorbitol dehydrogenase, FK, fructokinase; (**c**) SO, sorbitol oxidase, GK, glucokinase; (**d**) DH, quinate dehydrogenase; (**e**) MS, malate synthase, NADP-ME, NADP-malic enzyme, NAD-ME, NAD-malic enzyme; (**f**) CS, citrate synthase, GAD, glutamate decarboxylase; (**g**) LOX, lipoxygenases, HPL, hydroperoxide lyase; (**h**) ADH, alcohol dehydrogenase; (**i**) AAT, alcohol acyl-transferases; (**j**) ACX, acyl-CoA Oxidase; (**k**) CCD, carotenoid cleavage dioxygenases; (**l**) TPS, terpene synthase. Two capital letters in the top are the cultivars shown in Table 1. All data are expressed as means ± standard error of triplicate samples.

Nine enzymes involved in sugar metabolism were analyzed in apricot fruit during the whole development process (Figure 7a). With regard to enzymes involved in sucrose accumulation, significant increase of activities in sucrose synthase (SS) responsible for synthesis direction (SSthy) and sucrose phosphatesynthase (SPS) were found throughout the whole development process. A significant increase of activity in SS responsible for degradation direction (SSca) was also observed during the development process, although to a lesser extent than SSthy and SPS. Significantly higher levels of SSthy, SPS and SSca were found in LT than those in other cultivars. In addition to synthesis, sucrose can also be cleaved by neutral invertase (NI) and acid invertase (AI), no significant changes were found for these enzymes. Sorbitol can be inverted into fructose by sorbitol dehydrogenase (SDH), and a significant increase SDH enzyme activity was observed during the whole development period (Figure 7b). Fructose can be cleaved by fructokinase (FK), nevertheless, no significant change was found during ripening process (Figure 7b). In addition, sorbitol can be also oxidized into glucose by sorbitol oxidase (SO), and a significant increase was found during whole development period (Figure 7c). In contrast, glucose can be cleaved by glucokinase (GK), however, no significant change was observed in the enzyme activity. For all cultivars tested, no significant differences in the nine enzymes for sugar metabolism were observed between peels and pulps.

Six enzymes involved in organic acid metabolism were analyzed in apricot during the fruit development and ripening process. Quinate dehydrogenase (QH) is responsible for converting 3-dehydroquinate into quinate, and significant decrease was found in apricot during fruit development process (Figure 7d). With regard to malate, malate synthase (MS) is the key enzyme for synthesis, and a significant decrease in its activity was observed throughout the whole development process, which was consistent with the decrease of malate content (Figure 7e). Significantly higher levels of MS enzyme activity was found in YC fruit than in other cultivars. Malate can also be metabolized by NADP-malic enzyme (NADP-ME), and NAD-malic enzyme (NAD-ME), and a significant increase in these enzymes were found in cultivars tested in both peels and pulps, which leads to the decease of malate content during the development and ripening process (Figure 7e). Citrate synthase (CS) mainly is responsible for the citrate biosynthesis, and the activity was induced significantly during fruit development and ripening (Figure 7f). It is reported that glutamate decarboxylase (GAD) activity participates in regulating cytosolic pH, while no significant change in GAD activity was found in apricot throughout whole development process (Figure 7f). Similar to the enzymes of sugar metabolism, no significant differences in the six enzymes for organic acid metabolism were observed between peels and pulps.

Significant increase of lipoxygenases (LOX), hydroperoxide lyase (HPL), alcohol dehydrogenase (ADH), alcohol acyl-transferases (AAT), Acyl-CoA Oxidase (ACX) activities were found throughout the whole development period of apricot fruit (Figure 7g–j). Similar remarkable increase trend was observed in peels or pulps of apricot tested during fruit development process. A rapid significant increase in carotenoid cleavage dioxygenases (CCD) activity was found during fruit development process in both peels or pulps tested (Figure 7k), whereas the terpene synthase (TPS) activity decreased significantly during this process (Figure 7l). On the whole, the enzyme activities of aroma volatiles metabolism in peels were significantly higher than those in pulps of all cultivars tested.

### 2.7. Correlation between Enzymatic Capacities and Flavor Compound Accumulation

To search for possible links between enzymatic activities and flavor compounds accumulation, a Spearman’s correlation analysis was performed.

**Figure 8:**
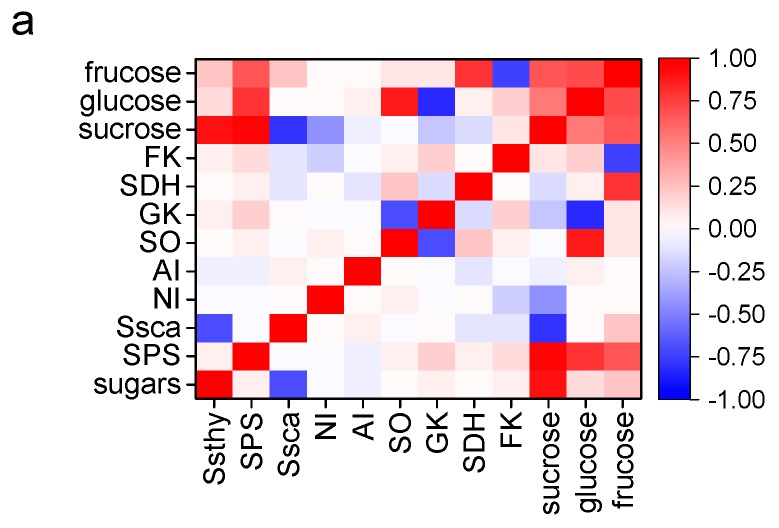

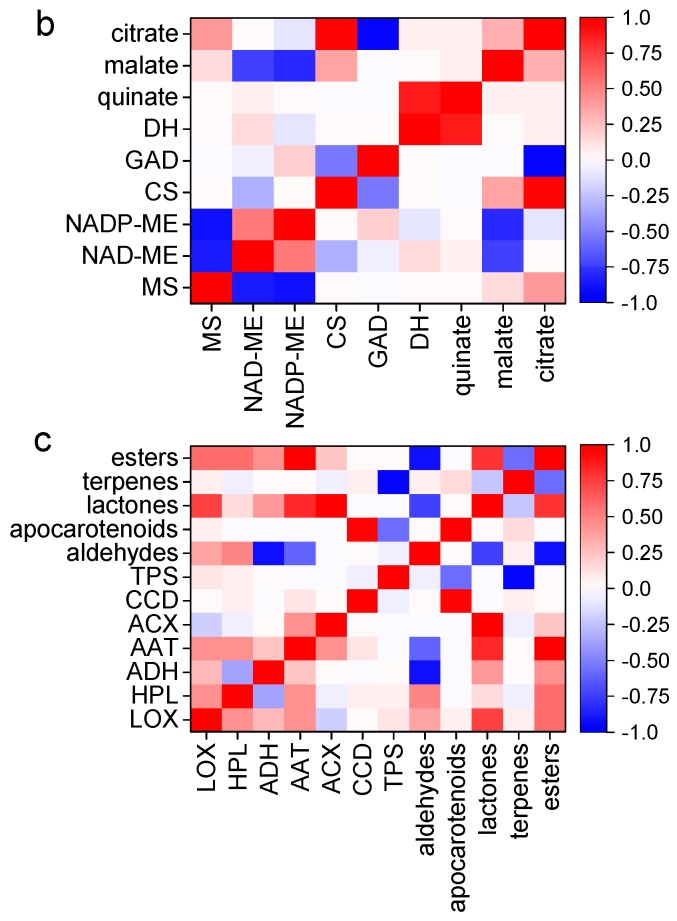
Visualization of Spearman’s correlations between flavor compound contents and metabolism enzyme activities during apricot fruit development. The square color corresponds to the correlation value as shown in the legend: blue represents a negative correlation, and red represents a positive correlation. The white squares correspond to non-significant correlations (*p* value > 0.01). (**a**) Correlation between enzyme activities of sugar metabolism and sugar contents: SSthy, sucrose synthase for synthesis direction, SPS, Sucrose phosphatesynthase, SSca sucrose synthase for degradation direction, NI, neutral invertase, AI, acid invertase, SDH, sorbitol dehydrogenase, FK, fructokinase, SO, sorbitol oxidase, GK, Glucokinase; (**b**) Correlation between enzymes activities of organic acid metabolism and organic acid contents: QH, quinate dehydrogenase, MS, malate synthase, NADP-ME, NADP-malic enzyme, NAD-ME, NAD-malic enzyme, CS, citrate synthase, GAD, glutamate decarboxylase; (**c**) Correlation between enzyme activities of aroma volatile metabolism and aroma volatile contents: LOX, lipoxygenases, HPL, hydroperoxide lyase, ADH, alcohol dehydrogenase, AAT, alcohol acyl-transferases, ACX, Acyl-CoA Oxidase, CCD, carotenoid cleavage dioxygenases, TPS, terpene synthase. All data are expressed as means ± standard error of triplicate samples.

As seen in Figure 8a, the contents of fructose, glucose and sucrose are significantly positively correlated with SPS activity (*p* < 0.01). Similarly, a significant positive correlation was observed between the contents of sucrose, total sugars and Ssthy activity (*p* < 0.01). In contrast, a significant negative correlation was found between the contents of sucrose, total sugars and Ssca activity. No other significant correlations were found between the enzyme activities of sugar metabolism and sugar accumulation. Regarding organic acids (Figure 8b), the content of malate was significantly positively correlated with MS activity, but negatively with NADP-ME and NAD-ME activities (*p* < 0.01). The content of citrate was significantly positively correlated with CS activity, but negatively with GAD activity (*p* < 0.01). As shown in Figure 8c, ester and lactone accumulation is significantly positively correlated with LOX, HPL, ADH, AAT and ACX activities (*p* < 0.01), however, significant negative correlations were found between the content of aldehydes and ADH activity. The contents of apocarotenoids and terpenes are significantly positively correlated with CCD and TPS activities, respectively.

## 3. Discussion

In the study, we firstly presented an analysis of sugars, organic acids, and aroma volatiles metabolism during apricot fruit development and ripening. The key taste and aroma components in apricot were identified by integrating chemical composition with consumers’ evaluation. The use of five cultivars removes the complex effect of genetic background, highlighting the common trends and revealing the possible accumulation mechanism of the main flavor compounds underlying development and ripening.

In general, the composition of sugars in fruits mainly depends on genetics; however, cultivation condition and environmental factors mainly influence the amount of total soluble sugars. Even the peak sugar levels at maturation or ripening and the sugar accumulation patterns and concentration differ between species and are regulated by fruit development. Sucrose in peach fruit increases until maturity [20]. Fructose and sucrose are the major soluble sugars whereas sorbitol was the major sugar alcohol in mature fruit. As apple fruit develops, fructose significantly increases, which results from sucrose that was transported from the leaves and newly synthesized sucrose, while the sorbitol decreases throughout fruit development, the occurrence of a glucose peak at about 6 weeks after boom [21]. In mango, the major sugars were identified as glucose, fructose and sucrose, and their contents increase during ripening; sucrose was found to be in the greatest concentration throughout ripening process [22]. Glucose and fructose significantly accumulate in sweet cherry during ripening. Fructose and glucose are the main sugars in the date plum persimmon fruit [23].

In the present study, we found that all sugars increased rapidly throughout the whole development process, and glucose and sucrose were the major sugars in pulps or peels of all cultivars tested. At the early stage glucose was the most abundant sugar, and as development proceeds, sucrose accumulation is enhanced significantly. At the late stage of fruit development, the content of sucrose exceeds that of glucose. The apricot cultivars tested mainly accumulated glucose during early development, whereas the fruit mainly accumulated sucrose during ripening, which suggested that the accumulation pattern of sugars in apricot fruit convert from glucose-predominated to sucrose-predominated during fruit development and ripening. In relation to the enzymes involved in sugar metabolism, the drastic increase in SSthy and SPS are responsible for the rapid increase of sucrose, which is consistent with the correlation analysis between enzyme activities and sugar accumulation. No significant changes in NI and AI enzymes were observed during the process, which showed that invertases is not important for sugar accumulation in apricot fruit, which is similar to the results found in apple [24], but different from peach in which NI is a clear candidate for mediating several aspects of sugar metabolism [8]. Even trace sorbitol was detected in apricot throughout the development and ripening period. A significant increase in glucose and fructose were found, which corresponded with the increase in SO and SDH enzymes activities. Therefore, it is possible that sorbitol is converted to glucose and fructose via the two enzymes, which suggests that the accumulation of these sugars mainly comes from sorbitol catalysis. However, no significant increases in GK and FK enzymes activities were observed in apricot fruit, which suggested that little glucose and fructose were converted into other sugars. These results and the data shown in the present work suggest that SS, SO and SDH are under tight developmental control and that they might play important roles in sugar accumulation in apricot fruit. Though there were no significant differences found between peels and pulps, as the sweetest sugar, fructose ratio was significantly higher in pulps than that in peels, which was consistent with that fact that the sweetness taste was stronger in pulps than that in peels. At the same time, the tissue difference may be due to high SDH enzyme activity. For cultivars, the sucrose content in DX, HY and BX were significantly higher than that in YC and LT, but the glucose content was significantly lower. On the whole, LT presented significantly higher fructose, glucose, sucrose and total sugars than other cultivars, which supports the evidence that LT is sweeter than other cultivars, and its high sugar content accounts for the high SPS, SSca and SDH enzymes activities.

In this study, almost all identified organic acids increased at first and then fell throughout fruit development and ripening process. Quinate, malate and citrate were the most abundant organic acids, similar to peach fruit [25]. At early stages, quinate and malate are the major organic acids, they occupied more than 85.18% of total organic acid, whereas the ratio was changed with the rapid increase of citrate at the maturation stage. The quinate, malate and citrate become the major organic acids at the ripening stage, these three organic acids accounted for over 95% of total organic acid. As the major organic acids in apricot, citrate, quinate and malate increased at fruit development stage and then fell slightly at the ripening stage, which is similar with the trend that found in peach maturation stage [20]. Quinate and malate declined at fruit maturation, however, the citrate decreased at the ripening stage, which resulted in high ratio of citrate to total organic acid. Citric and malic acids were found to be the major organic acids in apple. A large decrease in citric acid and a small reduction in malic acid were responsible for the loss of acidity [22], which lead to the result that malic acid is the main organic acid in mature apple. For citrus, quinic acid was the major organic acid whatever the fruit variety during the first 50 days of development, afterward, citric acid predominated in acidic varieties, while in acidless, malic acid exceeded it [26]. The major organic acids found in date plum fruit were citric and malic acids, which increased through the immature stages to maturity, and then decreased in the overripe fruit [27]. QH was the last enzyme responsible for quinate synthesis, the significant decrease of enzyme activity and quinate were concomitantly observed, which suggested that QH was the key enzyme for quinate accumulation. During fruit development, apricot undergoes a continuous accumulation of organic acids, which are used as respiratory substrates [28]. MS is the key enzyme for malate formation, while NADP-ME and NAD-ME are two key enzymes for malate oxidation by mitochondria [5]. The decrease in MS and increase in NADP-ME and NAD-ME were together responsible for the malate decrease throughout the development and ripening process, which is further confirmed by the correlation analysis between these enzyme activities and malate accumulation in this process (Figure 8b). CS was identified as the key enzyme for citrate synthesis, while citrate also can be metabolized in the GABA shunt pathway by GAD. The increase in CS enzyme activity mainly accounts for the increase of citrate while no increase of GAD enzyme activity was observed during the process, which indicates that the GABA shunt is not important for citrate accumulation in apricot fruit. Similarly, no significant content differences of organic acids were found between peels and pulps, but significantly higher citrate was found in pulps than that in peels. Even though citrate is not the sourest of the organic acids, citrate gives a stronger taste than quinate and malate, which may strengthen the integration of sourness taste of apricot pulps. However, as no CS enzyme activity difference was found between them, GABA shunt may have some contribution to the difference. During the ripening period, all cultivars shared a similar pattern of organic acids accumulation, however, the ratio of quinate, malate and citrate depended on cultivars. The ratio of malate, quinate, and citrate in DX and HY was 1:1:0.7, in HY, LT and BX was 2:1:1, which led to the acidity difference between cultivars.

Odor activity values (OAV) are usually used to identify the key aroma compounds in food, and in the present study a total of 18 aroma compounds were key components of apricot fruit by this method, which largely consistent with previous studies [29,30]. Among these compounds, their content and compositions presented different patterns during apricot fruit development and ripening. Aldehydes and terpenes decreased significantly during the whole development of apricot fruit, whereas lactones steeply increased with fruit ripening; these results are consistent with previous studies [18]. Seven key enzymes involved in aroma metabolism were analyzed in apricot during fruit development and ripening. In the fatty acid pathway, unsaturated fatty acids linoleic acid (18:2) and linolenic acid (18:3) can be cleaved into hydroperoxides by LOX, which are subsequently cleaved by HPL to form hexanal or nanonal. Aldehydes can then be reduced to the corresponding alcohols by ADH. AAT catalyzes the final linkage of an acyl moiety and an alcohol to form esters [31]. In the present study, all enzyme activities in the LOX pathway increased rapidly throughout fruit development and ripening process, which is consistent with the corresponding aroma products of the metabolic flux. The β-oxidation of fatty acid is considered as another pathway for lactones formation, the first enzyme acyl-CoA oxidase of this process is involved in lactone formation [32]. The accumulation of lactones, especially for γ-decalactone, kept consistent with ACX enzyme activities, which provide the further evidence for β-oxidation involved in lactones formation in fruit. Carotenoids can be cleaved into volatile apocarotenoids in fruit by CCD [33,34]. During apricot development and ripening, a significant increase in CCD enzyme activity was observed in apricot fruit and it was in agreement with the correlation analysis between apocarotenoid accumulation and CCD enzyme activity. In addition, light-colored cultivars contained abundant β-ionone and dihydro-β-ionone. For example, LT and BX had higher CCD enzyme activity than other cultivars, which indicates that the carotenoids in these cultivars may be cleaved into volatile apocarotenoids and suggests that this enzyme is important for conformation of color and aroma quality, and future studies will be required to further elucidate this contribution. TPS was considered the key enzyme for volatile monoterpenes, which was consistent with the decrease found in apricot fruit throughout the development and ripening process and the correlation analysis. Thus, TPS may also be involved in monoterpene biosynthesis in apricot fruit. Likewise, significantly higher enzyme activities of aroma volatile metabolism were found in peels than in pulps, which suggests that peels have more strong aroma volatile synthesis abilities than pulps have. The majority of aroma volatiles in peels were significantly higher than those in pulps, which was consistent with their OAVs of each compound and the changes of metabolic enzyme activities. Furthermore, the contents of β-ionone, β-damascenone and γ-decalactone in LT and BX were significantly higher than those in other cultivars, which suggested that white apricot presented stronger flowery and peach-like aroma than other cultivars. This change was consistent with CCD and ACX enzyme activities in LT and BX.

A PLSR model was developed with the aim of identifying the main variables influencing consumer acceptability. This procedure allowed a rapid assessment of relationships between the dependent variable (Y) and a set of potentially explanatory variables (X). To determine the variables that attribute to consumer acceptance of fruit, a PLSR model for apricot (including cultivar and development) was developed. In the present study, panel test was used to assess whether the changes in flavor compounds could directly affect aroma, taste and consumer acceptance of apricot fruit. We found that LT and BX fruit had a markedly higher aroma, sweetness, flavor rating and consumer acceptance than other cultivars, along with lower sourness. These results suggest that LT and BX present superior fruit flavor quality to other cultivars, and that consumer acceptance depends on predominant characteristic flavor compounds. The PLSR model was used to identify the main contributors to flavor quality and consumer acceptance, and the result showed that β-ionone, γ-decalactone, sucrose, citrate, sweetness, aroma and flavor were significantly positive with consumer acceptance, but markedly negative with malic acid, hexanal, (*E*,*Z*)-2,6-nonadienal and sourness, which were consistent with the changes of flavor compounds and organoleptic evaluation. Meanwhile, we found there were positive correlations between aroma, flavor and acceptability, which implied that flavor plays a central role in fruit quality and it triggers consumers’ acceptance or choice.

## 4. Materials and Methods

### 4.1. Fruit Materials Preparation

Apricot fruit were harvested from trees grown in an experimental orchard located in National Fruit Tree Germplasm Repository, Academy of Xinjiang Agricultural Sciences, Luntai, Xinjiang, China (45°19′ N, 86°03′ E). All experimental trees were planted at a 3 × 4 m spacing in 1998 in rows in a north-south orientation. Fertilization management and pest control was carried out according to standard practices and drip irrigation was used to supply fruit trees with water. Experimental design was a singletree plot complete randomized design with 10 individual trees as replications for every cultivar.

During the 2015 harvest season (from the 12 April to 30 July), a total of 240 fruits were picked from ten trees at the each stage on the basis of external color and size uniformity for each cultivar (Figure 1), and the specific sampling time for each cultivar was given in Table 1. After harvest, fruits were randomly divided into three replicates. After removing the pit and seeds, fruit were divided into peels and pulps by peeling knife, and cut into small cubes. Each replicate included 80 fruits. Among these fruits, twenty were used to determine the TA and TSS. Sixty were ground into a fine powder in liquid nitrogen using a freezer-mill (6750) apparatus (Glen Creston, Middlesex, UK), then the powder was stored at −80 °C until analysis. In the study, three replicates were performed for all chemical analyses.

### 4.2. Fruit Quality Traits Determination

Peel color at different developmental stages was measured using a Hunter Lab Mini Scan XE Plus colorimeter (Hunter Associates Laboratory, Inc., Reston, VA, USA). The CIE L*a*b*color scale was adopted, and the data were expressed as L*, a*, b*, C*, H, four random measurements per fruit were made and a mean value was obtained from ten fruits per replicate.

Flesh firmness was measured at the equator of the fruit using a penetrometer (Model: HL-300, Xianlin Non Detection Device Co., Ltd., Nanjing, China) with an 8 mm diameter head. Ten fruit were used as one replicate and two measurements were made on opposite sides of each fruit after removal of a 1 mm thick slice of peel, and three replicates were used for every time point samples.

TSS and TA measurements were conducted on juice samples collected from ten fruit for one replicate, and three replicates were used. TA measurements were performed by titrating 10 mL of juice with 0.2 M NaOH until reaching a pH of 8.2, and TA values were expressed as mmol L^−1^ H^+^. TSS values on the opposite parts of each fruit were measured with a hand-held refractometer (Model: B32T Brix Meter, Guangzhou Ruiqi Trade Co. Ltd., Guangdong, China).

The production of ethylene by the fruit was measured according to method described by Xi et al. [21]. Ten fruits as one replicate was placed in a sealed 1 L glass container for one hour and three replicates were used for every time point samples. Gas samples (1 mL) were then extracted from the containers and injected into an Agilent 7820A gas chromatograph (GC; Agilent Technologies, Inc., Santa Clara, CA USA) fitted with a stainless steel Supelco Porapak-Q column (2 m in length, o.d., 3.175 mm; mesh size, 80/100; Supelco, Bellefonte, PA, USA) and a flame ionization detector (FID). Nitrogen was used as the carrier gas. The column temperature was maintained at 80 °C, the injector temperature at 150 °C, and the detector temperature at 200 °C.

### 4.3. HPLC Analysis on Sugars and Organic Acids

Sugars and organic acids were extracted as described by Zhang et al. [35]. For each sample, 5.0 mL of cold ethanol (80%) was added to 2 g frozen powder. The sample was then incubated for 20 min in a 35 °C water bath and centrifuged at 10,000× *g* for 10 min. This extraction procedure was repeated three times and the supernatants were combined. The total volume was then adjusted to 25 mL with 80% ethanol. From this mixture, 1 mL was dried under a vacuum (Eppendorf Concentrate Plus, Hamburg, Germany) at 45 °C, and the residue was resuspended in 0.5 mL of distilled water and filtered through a 0.22 μm, 13 mm diameter syringe filter (Shanghaixingya Purification Material Factory, Shanghai, China). The filtered solution was then used for the sugar and organic acid analysis.

A chromatographic separation of sugars involved acetonitrile: water (80:20, *v*/*v*) as the mobile phase at a flow rate of 1.4 mL·min^−1^ with an Agilent ZORBAX Carbohydrate (4.5 μm, 4.6 mm × 250 mm) column (GL Sciences Inc., Torrance, CA, USA). Eluted peaks were detected with an SHODEX RI101 refractive index detector (JASCO International Co., Ltd., Tokyo, Japan). The data were analyzed with a Chromeleon^®^ 6.8 chromatography data system (Thermo Fisher Scientific Inc., Waltham, MA, USA).

The chromatographic separation used for organic acid detection employed (NH_4_)_2_HPO_4_ (50 mM, pH 2.7) as the mobile phase, with a flow rate of 0.5 mL·min^−1^, and the samples were injected onto an ODS C_18_ (4.6 mm × 250 mm) column (Beckman Coulter Inc., Brea, CA, USA). Organic acids were detected with a 2996 diode array detector (Waters Beckman Coulter Inc., Brea, CA, USA) at a wavelength of 210 nm. Soluble sugars and organic acids were quantified according to standard curves of authentic compounds. Extracts from three replicates tissue samples were analyzed. The data were analyzed with a Waters Empower system.

### 4.4. GC-MS Analysis

The concentrations of volatiles were determined according to our previously reported method with some modifications [36]. For each sample, 1.5 g frozen powder was homogenized with a saturated sodium chloride solution and held at 40 °C for 30 min. A solid-phase microextraction (SPME) needle with a 1 cm long fiber coated with 65 μm of Divinylbenzene/Carboxen/Polydimethylsiloxane (DVB/CAR/PDMS) fibers (Supelco Co., Bellefonte PA, USA) was used for volatile extraction.

A GCMS-QP2010 gas chromatograph-mass spectrometer system (Shimadzu Corporation, Kyoto, Japan) with an Rtx-5MS (Restek)-fused silica capillary column (5% diphenyl, 95% dimethyl polysiloxane) (0.32 mm, 30 m, 0.5 μm, J&W Scientific, Folsom CA, USA) was used for compound confirmation. The injection port temperature was 240 °C. The injection volume was 1 μL. Helium was used as the carrier gas at a rate of 1.0 mL·min^−1^. The GC oven temperature was held at 40 °C for 3 min, increased by 4 °C·min^−1^ to 250 °C, and then held for 5 min. Mass spectra were obtained by electron ionization (EI) at 70 eV and a scan range of 40–500 mass units. The detector, ion source and transfer line temperature were set to 150, 200 and 250 °C, respectively. Quantitative determination of compounds was calculated based on standard curves of authentic compounds.

The chromatograms and mass spectra were evaluated with GC-MS Postrun Analysis software (SHIMADZU, GC-MS-QP2010, Tokyo, Japan) The compounds were tentatively identified by comparing their mass spectra with the data system library (NIST 98), linear retention indices (LRI) reported in the literature and EI mass spectra with data from authentic compounds.

### 4.5. Flavor and Consumer’s Acceptance Determinations

Sensory evaluation was conducted as described by Altisent et al. [37] and Xi et al. [32]. Each apricot was sliced into four pieces were presented on one plate to taste. The tasting panel was composed of 40 consumers (62% women and 38% men aged between 21 and 58 years) who were habitual apricot consumers. Mineral water was used as a palate cleanser between samples. The sweetness, sourness, aroma, flavor, and consumer acceptance were assessed by consumers. Each consumer assessed all three samples at the same paper ballot and was asked to indicate his/her degree of liking/disliking using a 9-category hedonic scale (1—dislike extremely to 9—like extremely).

### 4.6. Determination of Enzymes Activities

Ssthy, Ssca, SPS, NI, AI, GK, FK, SO and SDH were determined by Desnoues et al. [4]. MS, NADP-ME, NAD-ME activities were determined described by Lara et al. [38]. DH was determined according to Van Kleef and Duine [39]. CS, MS, GAD activities were determined by Borsani et al. [8] with some modifications. GAD activity was determined according to Snedden [40] with some modifications. LOX, HPL, ADH, AAT were determined described by Ortiz et al. [41]. The activity of ACX was measured by Xi et al. [32]. CCD activity was conducted according to Simkin et al. (2004). TPS activity was measured according to Abbott et al. [42]. In all cases, one activity unit (U) was defined as the variation in one unit of absorbance per minute. Each determination was done in triplicate, and results were expressed as specific activity (U/mg protein).

### 4.7. Statistical Analysis

All data are expressed as the means ± standard error of three replicates. A statistical analysis was performed using SPSS v19.0 software (SPSS Inc., Chicago, IL, USA). Significant differences among the samples were calculated using one-way ANOVA followed by Duncan’s multiple-range test at the 5% level (*p* ≤ 0.05). The PLSR were run to correlate sugars, organic acids, aromas and sensory attributes, as X variables, to consumer acceptability, the Y variable, to find the variables that had the most weight for discriminating consumer-preference fruit. Analyses were carried out at S4 and S5 of apricot ripening. The flavor compounds and their codes are in Table 2. The sensory parameters are in Table 3.

## 5. Conclusions

Overall, sucrose and glucose were the major sugars in apricot. All sugars increased rapidly throughout the maturation and ripening process, the accumulation pattern of sugars in apricot converted from glucose-predominated to sucrose-predominated during fruit development and ripening. SS, SO and SDH are under a tight developmental control and might play important roles in sugar accumulation of apricot fruit. Almost all organic acids identified increased at first and then fell throughout whole development process. Quinate, malate and citrate were the most abundant organic acids. At early development stage, quinate and malate are the major organic acids, with the rapid increase of citrate at the maturation stage; quinate, malate and citrate become the major organic acids at the ripening stage. A total of 18 aroma compounds were measured and the crucial components of apricot fruit were found to be γ-decalactone and β-ionone. Aldehydes and terpenes decreased significantly during apricot development and ripening, whereas lactones steeply increased with fruit ripening. β-Ionone, γ-decalactone, sucrose and citrate are the key flavor components contributing to the consumer’s acceptance in apricot fruit. Especially in light colored cultivars, volatile apocarotenoids such as β-ionone have important role in consumer preference, and CCD may be involved in formation of these compounds. These results provide important information for flavor quality regulation of fruit apricot and consumer-oriented breeding.

## Figures and Tables

**Table 1:** Apricot cultivars used in the present study and the sampling time for each stage.

No.	Cultivars	Abbreviation	Repository No. ^1^	Color Character		DAB ^2^				
				peel	pulp	S1	S2	S3	S4	S5
1	Danxing	DX	XD076	red	orange	21	32	56	74	82
2	Hongyuxing	HY	XD117	red	orange	21	32	61	74	82
3	Yechengheiyexing	YC	XD018	orange	white	21	27	61	91	91
4	Luntaixiaobaixing	LT	XD021	white	light yellow	21	27	57	65	74
5	Baixing	BX	XD094	white	white	21	27	61	88	91

^1^ The united national number of cultivars; ^2^ DAB, Days after blossom.

**Table 2:** Orthonasal odor thresholds (OOT) and odor activity values (OAV) of apricot aroma compounds.

No.	Aroma Compound	Code	OOT ^1^	Aroma Quality	OAV ^2^									
					Peel					Puip				
					DX	HY	YC	LT	BX	DX	HY	YC	LT	BX
1	hexanal	nh	2.4	Green, grassy	34.03	25.97	29.03	24.72	18.33	10.56	4.86	14.86	7.50	8.19
2	(*Z*)-3-hexenal	Zh	0.12	Green, grassy	5.42	3.17	2.58	1.75	19.75	1.33	0.92	1.17	1.17	5.42
3	(*E*,*Z*)-2,6-nonadienal	EZ26n	0.03	Cucumber-like	148.33	78	39	59.33	48.33	49	41	21.67	14.33	18.67
4	β-damascenone	bD	0.002	Baked apple-like, grape juice-like	45	40	55	180	145	26	35	15	130	85
5	β-ionone	bI	3.5	Flowery, violet-like	111	91.43	97.14	506	434	51.43	40	31.43	314	280
6	dihydro-β-ionone	DbI	5	Fresh rosy note	7.40	5.20	5.01	20.80	19.40	1.60	2.20	1.80	11.60	9.20
7	γ-octalactone	gOL	6.5	Coconut-like	10.31	8.12	6.62	10.92	8.31	7.23	5.85	5.85	7.23	5.23
8	δ-octalactone	dOL	0.4	Coconut-like, sweet odor	47.50	52.50	40	65	42.50	27.50	22.50	22.50	30	20
9	γ-decalactone	gDL	1.1	Peach-like, coconut-like	281	210	155	319	213	225	146	181	252	160
10	δ-decalactone	dDL	53	Coconut-like	2.16	5.04	3.36	5.21	4.42	2.32	2.17	1.26	1.55	1.26
11	γ-dodecalactone	gDOL	0.43	Peach-like	66.57	155	103	160	136	55.81	44.19	37.21	52.33	41.86
12	β-myrcene	bM	1.2	Hop-like, geranium-like	10.83	9.67	9.33	13.67	11.17	5.83	4.33	3.17	6.17	3
13	linalool	Li	6	Citrus-like, bergamot-like	57.50	14.50	11.17	20.67	20.67	14.50	9	9.33	9.33	7.50
14	α-terpineol	aT	0.33	Lilac-like, peach-like	103	72.73	60.61	54.55	39.39	42.42	30.30	15.15	15.15	12.12
15	geraniol	Ge	1.1	Rose-like, citrus-like	24.55	23.18	20.45	14.09	5	15.46	9.09	7.27	6.82	5
16	limonene	Lim	10	Citrus-like	8.60	7	6.50	3.80	4.5	2.3	1.5	1.2	1.1	1.5
17	hexyl acetate	He	2	A mild sweet odor	102	84	93	183	78	28	18	22.50	38	32
18	(*Z*)-3-hexenyl acetate	Z3He	3.9	Green; banana-like	6.15	7.95	5.90	12	4.9	5.4	3.9	3.3	4.4	2.8

^1^ Odour thresholds used to calculate the OAV values were obtained from the Leffingwell web page (www.leffingwell.com). When a range of values was found, the lowest value was used; ^2^ OOT presents the odor thresholds in water (μg/L).

**Table 3:** Global acceptability and sensory attributes of apricot fruit of stage 4 (S4) and stage 5 (S5).

Sensory Attributes	S4					S5				
	DX	HY	YC	LT	BX	DX	HY	YC	LT	BX
Sweetness	5.61	4.93	4.82	6.87	6.14	8.42	6.78	6.23	8.77	7.53
Sourness	5.49	4.46	4.23	4.92	4.36	3.44	3.82	2.17	2.54	3.36
Aroma	6.21	5.78	5.24	7.36	6.89	7.63	6.25	6.84	8.79	8.13
Flavor	6.54	5.08	5.57	7.54	7.21	7.91	6.45	6.22	8.56	7.81
Acceptability	6.93	5.74	5.36	8.12	7.76	8.13	7.17	7.60	9.03	8.42

## Supplementary Materials

Supplementary materials can be found at http://www.mdpi.com/1422-0067/17/7/998/s1.

## Abbreviations

TA	titratable acidity
TSS	total soluble solid
S1	stage 1
DX	danxing
HY	Hongyu
YC	Yechengheiyexing
LT	luntaixiaobaixing
BX	baixing
FW	fresh weight
OOT	orthonasal odor thresholds
OAV	odor activity values
PLSR	partial least squares regression
SSthy	sucrose synthase for synthesis direction
SPS	sucrose phosphatesynthase
SSca	sucrose synthase for degradation direction
NI	neutral invertase
AI	acid invertase
SDH	sorbitol dehydrogenase
FK	fructokinase
SO	sorbitol oxidase
GK	glucokinase
QH	quinate dehydrogenase
MS	malate synthase
NADP-ME	NADP-malic enzyme
NAD-ME	NAD-malic enzyme
CS	citrate synthase
GAD	glutamate decarboxylase
LOX	lipoxygenases
HPL	hydroperoxide lyase
ADH	alcohol dehydrogenase
AAT	alcohol acyl-transferases
ACX	Acyl-CoA Oxidase
CCD	carotenoid cleavage dioxygenases
TPS	terpene Synthase
