# Supplementary material for: Profiling Taste and Aroma Compound Metabolism during Apricot Fruit Development and Ripening
Source: Int J Mol Sci. 2016 Jun 24;17(7):998. doi: 10.3390/ijms17070998 (PMC4964374; doi:10.3390/ijms17070998)
Supplement: Supplementary file 1 [file ijms-17-00998-s001.pdf]

Supplementary Materials: Profiling Taste and Aroma Compound Metabolism during Apricot Fruit Development and Ripening

Wanpeng Xi, Huiwen Zheng, Qiuyun Zhang and Wenhui Li

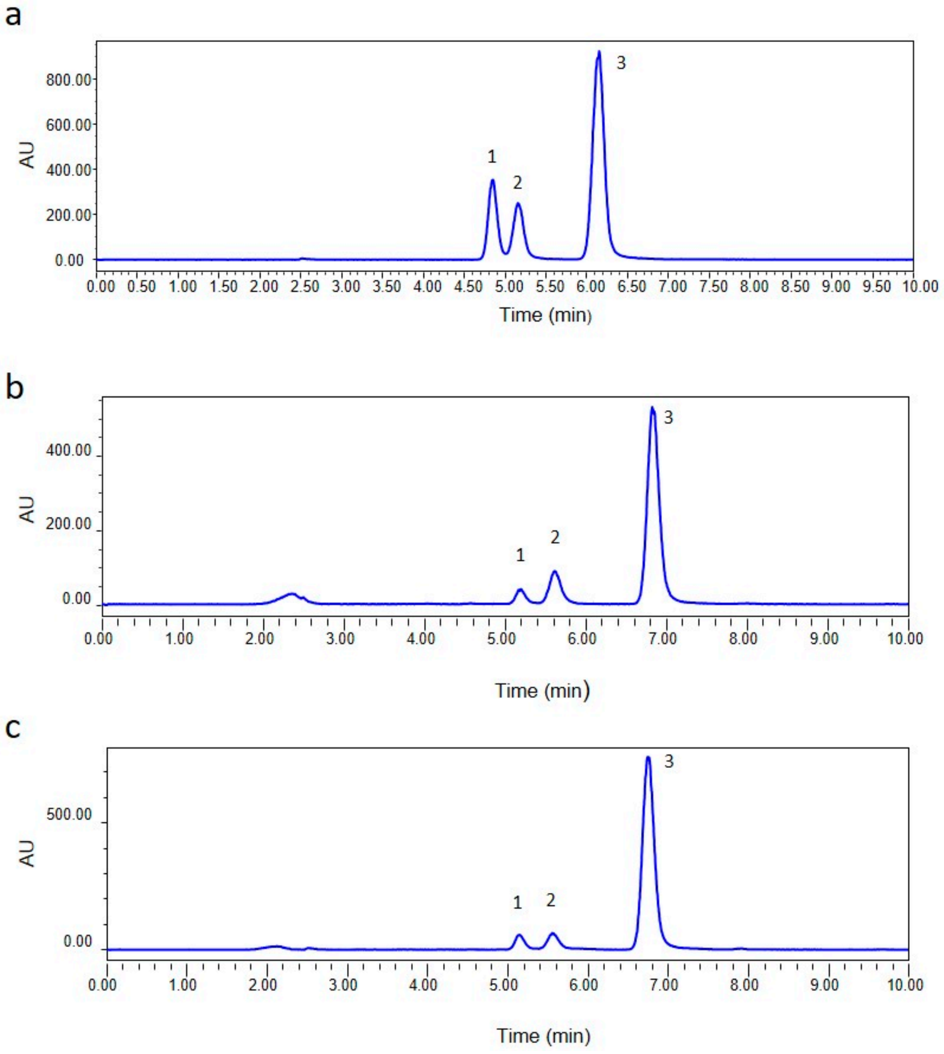

Figure S1. Sugars HPLC chromatogram of apricot fruit. Peaks (1) Fructose (2) Glucose (3) Sucrose. (**a**) sugars mixture standards; (**b**) sugars for SG peel of S5; (**c**) sugars for SG pulp of S5.

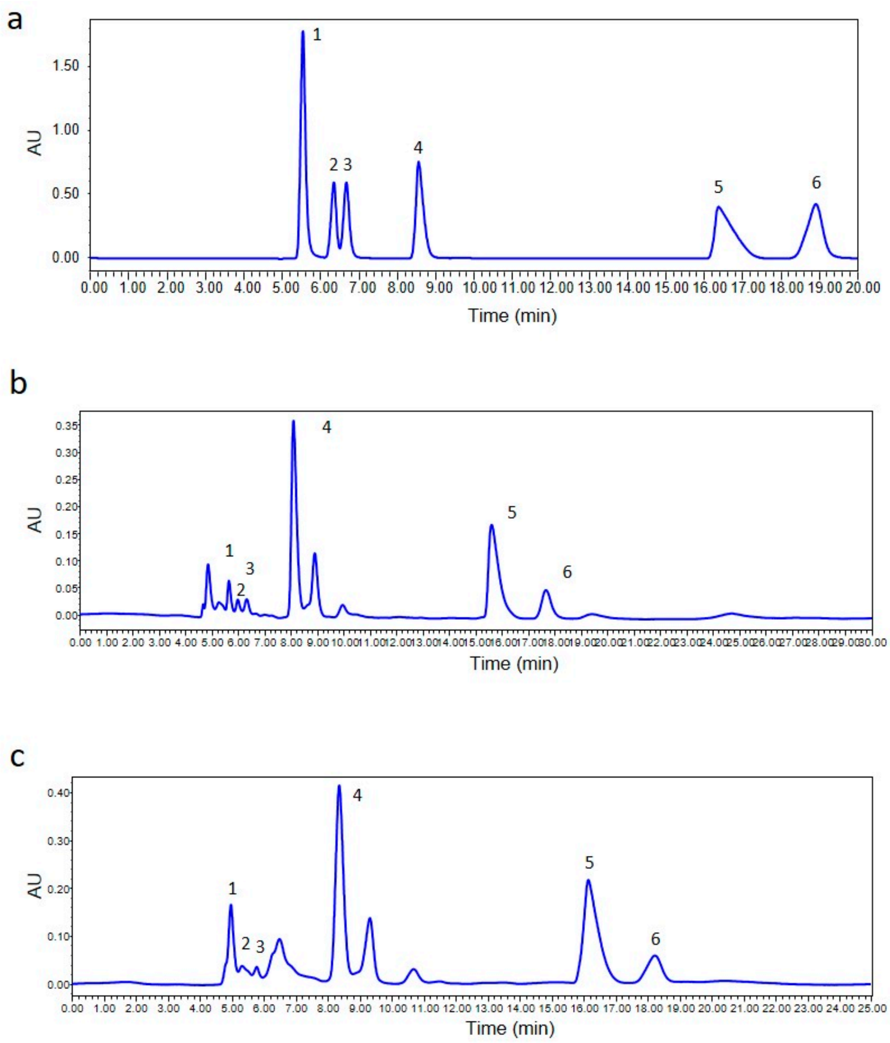

Figure S2. Organic acids HPLC chromatogram of apricot fruit. Peaks (1) oxalic acid (2) tartaric acid (3) quininic acid (4) malic acid (5) citric acid (6) fumaric acid. (**a**) organic acids mixture standard; (**b**) organic acid for YC peel of S4; (**c**) organic acid for YC pulp of S4.

Table S1. Chroma values of apricot fruit during development and ripening.

Cultivars	L*					a*					b*					C*					H				
	**S1**	**S2**	**S3**	**S4**	**S5**	**S1**	**S2**	**S3**	**S4**	**S5**	**S1**	**S2**	**S3**	**S4**	**S5**	**S1**	**S2**	**S3**	**S4**	**S5**	**S1**	**S2**	**S3**	**S4**	**S5**
DX	54.52	57.13	61.56	62.61	55.49	20.28	18.13	14.41	8.87	17.97	39.02	37.67	38.27	39.99	43.83	43.98	42.27	41.06	42.26	47.67	117.46	116.92	110.21	76.26	67.36
HY	54.97	55.83	61.53	65.08	65.11	−16.04	−14.77	−13.30	11.01	12.78	34.44	31.68	32.74	38.82	42.51	37.99	34.84	35.35	52.69	45.14	114.91	114.94	112.05	72.65	72.57
KE	47.02	47.00	48.32	60.11	60.66	−17.08	−17.37	−16.65	−0.36	8.20	30.83	29.09	29.08	48.23	48.48	35.26	33.88	33.51	48.33	49.33	118.94	120.86	119.85	90.67	118.02
AK	50.02	50.90	52.19	68.35	60.28	−21.98	−21.76	−19.02	-4.97	4.04	40.07	39.12	35.71	50.10	47.02	45.56	44.77	40.47	50.44	45.30	118.79	119.15	118.11	95.76	84.25
KZ	52.59	53.34	52.69	67.75	65.24	−21.64	−21.30	−19.05	−1.92	1.39	42.37	39.49	37.48	48.47	47.29	47.57	44.88	41.83	48.82	47.43	117.07	118.45	116.93	92.86	88.08
SG	49.84	51.13	55.12	66.47	67.75	−16.66	−17.02	−16.06	−8.75	1.91	31.53	31.76	32.43	40.59	48.87	35.67	36.04	36.21	41.80	48.95	117.80	117.65	116.36	101.96	87.77
SL	47.04	47.68	57.11	68.35	59.49	−20.71	−19.58	−17.10	−2.35	−1.01	34.38	32.09	37.59	51.09	41.01	40.37	37.61	41.34	51.41	41.06	125.31	121.45	114.44	92.70	91.74
YC	51.33	56.99	48.65	69.48	69.85	−21.83	−21.97	−18.42	−10.30	−4.04	41.12	43.13	32.85	41.82	42.51	46.71	48.41	37.63	43.15	42.85	117.88	117.04	123.72	104.28	95.23
BX	48.58	50.91	54.63	67.10	63.17	−21.23	−21.40	−19.24	−3.06	−1.58	37.80	36.98	36.72	45.86	44.36	43.35	42.72	41.47	46.12	44.81	119.33	120.10	118.30	93.71	91.69
LT	49.19	51.69	59.03	65.24	66.26	−19.84	−20.76	−15.62	−13.08	−1.86	37.99	38.80	39.86	41.27	43.30	42.95	43.55	42.90	43.33	43.37	117.95	118.57	111.59	107.48	92.56

Table S2. Identified aroma compounds in apricot fruit in the study [a,b].

No.	Compounds	Identification
1	hexanal	LRI, MS, Std
2	(*Z*)-3-hexenal	LRI, MS, Std
3	(*E*)-2-nonenal	LRI, MS, Std
4	trans-4,5-epoxy-(*E*)-2-decenal	LRI, MS, Std
5	acetaldehyde	LRI, MS, Std
6	(*E*,*Z*)-2,6-nonadienal	LRI, MS, Std
7	(*E*)-2-hexen-1-ol	LRI, MS, Std
8	2-methoxyphenol	LRI, MS, Std
9	hexanol	LRI, MS, Std
10	(*Z*)-3-hexen-1-ol	LRI, MS, Std
11	butanol	LRI, MS, Std
12	(*E*)-2-hexenyl acetate	LRI, MS, Std
13	hexyl acetate	LRI, MS, Std
14	(*Z*)-3-hexenyl acetate	LRI, MS, Std
15	butyl acetate	LRI, MS, Std
16	3-methylbutyl acetate	LRI, MS, Std
17	pentyl acetate	LRI, MS, Std
18	Heptyl acetate	LRI, MS, Std
19	β-Damascenone	LRI, MS, Std
20	β-Ionone	LRI, MS, Std
21	Dihydro-β-ionone	LRI, MS, Std
22	3-Hydroxy-7,8-dihydro-b-ionone	LRI, MS, Std
23	3-Hhydroxy-5,6-epoxy-b-ionone	LRI, MS, Std
24	γ-hexalactone	LRI, MS, Std
25	γ-octalactone	LRI, MS, Std
26	δ-octalactone	LRI, MS, Std
27	γ-nonalactone	LRI, MS, Std
28	γ-decalactone	LRI, MS, Std
29	δ-decalactone	LRI, MS, Std
30	γ-undecalactone	LRI, MS, Std
31	γ-dodecalactone	LRI, MS, Std
32	β-myrcene	LRI, MS, Std
33	linalool	LRI, MS, Std
34	α-terpineol	LRI, MS, Std
35	geraniol	LRI, MS, Std
36	Limonene	LRI, MS, Std
39	eugenol	LRI, MS, Std
37	α-cyclocitral	LRI, Std
38	Inalool hydrate	LRI, MS, Std
39	4-terpinol	LRI, MS, Std
40	γ-terpinene	LRI, MS, Std
41	hexanoic acid	LRI, Std
42	butanoic acid	LRI, Std
43	acetic acid	LRI, Std
44	2-methylbutanoic acid	LRI, Std
45	pentanoic acid	LRI, MS, Std
46	3-methylbutanoic acid	LRI, MS, Std

[a] LRI, linear retention index calculated on DB-WAX capillary column; [b] Identification: methods of identification; LRI (linear retention index), MS tent. (tentatively identified by MS), Std (chemical standard). When only MS or LRI is available for the identification of a compound, it must be considered as an attempt of identification.
